# Acupuncture and moxibustion therapy for cognitive impairment: the microbiome–gut–brain axis and its role

**DOI:** 10.3389/fnins.2023.1275860

**Published:** 2024-01-11

**Authors:** Jiatian Shi, Xinyue Zhang, Jianhua Chen, Ruishi Shen, Huashun Cui, Huangan Wu

**Affiliations:** ^1^Department of Acupuncture and Moxibustion, Shuguang Hospital Affiliated to Shanghai University of Traditional Chinese Medicine, Shanghai, China; ^2^Department of Mental Health, Shanghai Mental Health Center, Shanghai, China; ^3^Department of Acupuncture and Moxibustion, Yueyang Hospital Affiliated to Shanghai University of Traditional Chinese Medicine, Shanghai, China

**Keywords:** microbiome–gut–brain (MGB) axis, cognitive impairment, acupuncture and moxibustion therapy, neuroendocrine–immune system, intestinal microbiota, inflammation

## Abstract

Cognitive impairment poses a significant burden on individuals, families, and society worldwide. Despite the lack of effective treatment strategies, emerging evidence suggests that the microbiome–gut–brain (MGB) axis may play a critical role in the pathogenesis of cognitive impairment. While targeted treatment is not yet comprehensive, recently, acupuncture and moxibustion therapy has participated increasingly in the treatment of degenerative diseases and has achieved a certain therapeutic effect. In this review, the possible mechanisms by which acupuncture and moxibustion therapy may improve cognitive impairment through the MGB axis are reviewed, including regulating gut microbial homeostasis, improving intestinal inflammation mediated by the neuroendocrine–immune system, and enhancing intestinal barrier function. We also discuss common acupoints and corresponding mechanism analysis to provide insights into further exploration of mechanisms that target the MGB axis and thereby intervene in cognitive impairment.

## 1 Introduction

The global aging process has led to an increase in the elderly population worldwide, and aging-related cognitive impairment (CI) has become a significant global health challenge due to its rising mortality and socioeconomic burdens. In rural China, the positive rate of cognitive impairment among elderly individuals aged 65 years and older is estimated to be 42.9% (Wang J. et al., 2020; Wang L. et al., 2020). In 2015, the estimated global economic burden of dementia was USD$ 818 billion, projected to double by 2030s (Pratchett, 2015). Despite innovative knowledge about the pathogenesis of cognitive impairment (especially Alzheimer’s disease, one of the most common causes of acquired CI), effective drugs are still lacking. Acupuncture and moxibustion therapy, guided by traditional Chinese medicine theory, is characterized by multi-target, multi-pathway, and multi-dimensional effects, making them simple to operate and easy to accept. In recent years, acupuncture and moxibustion therapy has been gradually accepted and widely used in basic and clinical studies as an alternative treatment in combination with drugs to treat CI. However, the mechanisms behind this have not been conclusively determined, and further research is warranted to refine relevant theories.

The term ‘gut–brain axis (GBA)’ first appeared in the 1980s (Khodabakhsh et al., 2021), and the theory of the microbiome–gut–brain (MGB) axis has been increasingly mentioned during the past decade. Bidirectional communications within the MGB axis integrate peripheral gastrointestinal function with cognition via neuroimmune–endocrine mediators (Kesika et al., 2021), making a difference in clinical practice through the association between dysbiosis and central nervous disorders (e.g., cognitive impairment) and functional gastrointestinal disorders. Therefore, new therapeutic strategies should be developed to prevent or alleviate cognitive impairment, and treatments targeting the MGB axis may be an innovative one. However, further research is needed on the correlation between the axis and cognitive impairment. Although several studies have investigated the improvement and prevention of cognitive function by acupuncture and moxibustion therapy associated with the MGB axis, a comprehensive review of these studies has not been conducted. Therefore, this review aims to highlight the impact of the MGB axis on cognitive function and explore the potential use of acupuncture and moxibustion therapy targeting this axis as cognitive improvers.

## 2 How does the MGB axis affect cognitive dysfunction?

The MGB axis is a bidirectional interactive neuroendocrine–immune network comprising the central nervous system (CNS), the neuroimmune and neuroendocrine system, the autonomic nervous system (ANS), and the gut microbiota, as shown in Supplementary Figure 1. The role of GBA is to monitor and integrate gut functions as well as to link emotional and cognitive centers of the brain with peripheral intestinal functions (Carabotti et al., 2015), which contributes to the proper maintenance of gastrointestinal homeostasis. Among them, microorganism residents in our gut are involved in a variety of regulatory and pathogenic processes in immune activation, intestinal permeability, enteric reflex, and entero-endocrine signaling. Studies have shown that dysregulation or disruption of the MGB axis may be one of the pathological mechanisms of cognitive impairment. For example, intestinal flora dysbiosis may lead to disruption of hormonal regulation and glucose homeostasis, which, in turn, leads to weight gain and insulin resistance. Excessive immune response would then be induced by intestinal microecological disturbances and damaged intestinal mucosal barrier, leaving the organism in a state of chronic low inflammation. Following the induction, central systems at all levels would independently or cooperatively relay information to CNS and cause apoptosis and degeneration of brain cells in the cerebral cortex or hippocampus, ultimately resulting in cognitive impairment. By analyzing relevant reviews and studies, we believe that the network of the MGB axis leads to the onset of cognitive impairment through several aspects, including gut microflora dysbiosis, immune system-mediated intestinal inflammation, metabolic disorders, and intestinal barrier destruction.

### 2.1 Alterations in microbiota species and diversity

Multiple experimental and clinical studies revealed that alterations in microbiota species and abundance are directly associated with the development of cognitive impairment (Sampson et al., 2016), including Alzheimer’s disease (AD) (Fox et al., 2019). Poole et al. 2013 pointed out that AD patients exhibit a greater abundance of pathogenic microbes, overall bacterial load, and LPS in the brain, while lower levels of Proteobacteria and greater levels of Actinobacteria. In addition, one recent systematic review indicated that gut microbes including *Bacteroides vulgatus* and *Campylobacter jejuni* have an influence on cognitive function in patients with AD by altering glutamate metabolism (Chang et al., 2020) and activating the vagal pathway. Administration of *E. coli* and subsequent alterations in the gut microbial composition in mice resulted in memory impairments (Cussotto et al., 2018). Significant relationships between microbiota diversity and enhanced cognitive flexibility as well as executive function have already been observed (Tooley, 2020). The decrease in microbial diversity could affect normal immunity (Lach et al., 2018), leading to increased peripheral inflammation and barrier permeability, all of which may perturb brain homeostasis and ultimately contribute to the pathogenesis of cognitive disorders. Studies show that the lack or loss of biodiversity in the human gastrointestinal tract has been associated with aging, metabolic inflammatory diseases (MIDs), and disorders of the brain, including neurodegenerative disorders (Fox et al., 2019).

### 2.2 Inflammatory responses mediated by the neuroendocrine and immune systems

The ‘neuroendocrine–immune network’ theory was first proposed by Prof. Besedovsky (Cui et al., 2021) in 1977, suggesting that there may be complex interactions among the neural, endocrine, and immune systems to maintain stability and improve body function through feedback regulation. Increasing evidence suggests that alterations in the immune system are associated with cognitive dysfunction (Bioque et al., 2021). Chronic inflammation, together with impaired immune regulatory function, often precedes cognitive decline by decades in many neurological disorders (Fox et al., 2019). Severance et al. proposed that the increased inflammation and gastrointestinal dysfunction observed in schizophrenic patients might be attributed to overactivated inflammatory responses along the GBA that lead to CNS disorders and psychological changes (Severance et al., 2013). For example, pro-inflammatory cytokines and the immune mediators produced by dysbiosis of the intestinal microorganisms may reach the CNS by activating the HPA system (Cussotto et al., 2018), which is considered the core stress efferent axis that is predominantly involved in memory and emotional responses (Arneth, 2018). In addition, intestinal flora could also regulate the release of neurotransmitters (Cussotto et al., 2018) to communicate with the CNS through the vagus nerve. For example, the probiotic *L. reuteri* was shown to enhance wound healing in mice by increasing central levels of oxytocin through a vagal pathway (Poutahidis et al., 2013). Moreover, gut microbiota produces different neurotransmitters such as dopamine, noradrenaline, and ɣ-aminobutyric acid (GABA), which influence hypothalamic function and the major neuroendocrine axes in the host through mediating the neuroendocrine function (Rooks and Garrett, 2016), thus altering behavior and brain neuro-chemistry. The results of another study revealed that the acidophilus probiotic was able to alleviate learning and memory-associated injuries in Alzheimer’s rats by reducing mitochondrial dysfunction induced by neurotrophic D-gal and AlCl3, which could be associated with its antioxidant properties. Additionally, the researcher indicated that the gut microbiota contributes to alteration in brain function through increasing brain-derived neurotrophic factor (BDNF)-mediated hippocampal experience-dependent learning (Arneth, 2018). For example, germ-free (GF) mice exhibit hyper-activation of the HPA axis and reduced the BDNF expression levels in the cortex and hippocampus (Cussotto et al., 2018), affecting synaptic plasticity in the hippocampus and brain function. However, a change in microbiota composition with the probiotics leads to an increase in BDNF expression, which regulates different aspects of brain activities and cognitive functions (Carabotti et al., 2015).

### 2.3 Endocrine and metabolic disorders

Several signaling molecules such as cerebrointestinal peptides released from enteroendocrine cells (EECs), modulated by the gut microbiota, have significant endocrine and metabolic functions and are able to communicate with the brain (Carabotti et al., 2015). For example, cholecystokinin (CCK), a peptide hormone in the gut and also a neuropeptide in the brain, could influence learning and memory function through CCK-A receptors (mnemonic effects) and CCK-B receptors (amnestic effects) (Bioque et al., 2021). Liraglutide, a GLP-1 receptor agonist, has been proven to promote neurogenesis as well as to prevent apoptosis and oxidation in the parietal cortex, hypothalamus, and medulla, with preliminary clinical evidence in improving cognition and preventing cognitive decline (Camkurt et al., 2018).

Obesity and type-2 diabetes mellitus (T2DM) are each independent risk factors for cognitive impairment and dysregulation of energy metabolism such as blood glucose and lipids predispose to CI through central metabolism and inflammation. Adiponectin is a protein released from adipose regulating energy expenditure, the perturbations of which may contribute to the development of CI through inflammatory pathways and metabolic changes, including insulin sensitivity dysregulation, mitochondrial dysfunction, and downregulation of BDNF (Wennberg et al., 2016). Moreover, as one of the main products of bacterial metabolism, SCFAs could directly affect brain function and behavior mainly by affecting the maturation and function of microglia (Erny et al., 2015). For example, butyrate-producing bacteria could affect host metabolism by modifying glucose production, the lower abundance of which is associated with higher T2DM risk (Fox et al., 2019).

## 3 Can acupuncture and moxibustion therapy improves cognitive impairment?

Acupuncture and moxibustion therapy is a therapeutic method with simple operation, is easy to accept under the guidance of traditional Chinese medicine theory, and is characterized by multi-target, multi-pathway, and multi-dimensional effects. In recent years, acupuncture and moxibustion as an alternative treatment in combination with drugs to treat cognitive impairment has been gradually accepted and widely used in a number of basic and clinical studies (Huang et al., 2020; Wu et al., 2023). Acupuncture signals are recognized as a potent form of sensory stimulation that ascends mainly through the spinal ventrolateral funiculus to the brain so as to improve cognitive abilities through the regulation of neuroplasticity (Zhao, 2008; Ji et al., 2021). Zhao, (2008) and Ji et al., (2021) have also pointed out that acupuncture can effectively prevent memory impairment by affecting hippocampal synaptic plasticity (Su et al., 2020). There is a meta-analysis of randomized controlled trials indicating that acupuncture-related treatments lasting for at least 6 weeks effectively improved the cognitive function of AD patients (Lin et al., 2022). In addition, moxibustion demonstrated statistical differences in enhancing MCI patients’ general cognitive function (Yin et al., 2022). Several systematic reviews (Zhang et al., 2019, 2021) revealed that acupuncture could improve motor and language function and daily living activities. Recently, the mechanisms of the MGB axis have attracted increased interest, which may support some data to clarify how acupuncture and moxibustion therapy improve cognitive impairment. We would then comprehensively analyze the possible mechanisms behind it, mainly through regulation of intestinal flora, inhibition of inflammatory state, and improvement of metabolism and intestinal barrier.

### 3.1 Acupuncture and moxibustion therapy restores the stabilization of intestinal microecology by regulating intestinal flora diversity

Lots of evidence (Song et al., 2019) has confirmed that the decrease in gut microbial diversity and changes in community composition and abundance are closely associated with the pathogenesis of many gastrointestinal disorders (Liu G.-H. et al., 2022). For instance, the diversity of intestinal flora in ulcerative colitis (UC) mice were shown to be significantly decreased than that of healthy mice (Wei et al., 2019). One study (Wei et al., 2019) shows that electroacupuncture (EA) and moxibustion treatment on ST36 and CV4 has the ability to restore the diversity and compositions of the intestinal flora of UC mice through improving their alpha diversity indices and beta diversity distributions, which stabilizes the structure of intestinal flora. Studies also indicated that moxibustion at Guanyuan (RN4) could increase the diversity and abundance of intestinal probiotics in elder rats (Ouyang et al., 2022).

### 3.2 Acupuncture and moxibustion therapy regulates multi-system disorders

#### 3.2.1 Improvement of intestinal inflammation

##### 3.2.1.1 The first way is regulating intestinal flora and its metabolites

Probiotics, which are beneficial microorganisms with antioxidant and immunomodulatory activities, play an essential role in maintaining intestinal ecological homeostasis (Ouyang et al., 2022). Acupuncture and moxibustion therapy have the ability to regulate the inflammatory response by harmonizing the balance between anti-inflammatory probiotics and pro-inflammatory pathogenic bacteria to maintain the stability of the internal environment. For example, Lactobacillus and Bifidobacteria could protect the host from inflammation by inhibiting toll-like receptor 4 expression as well as downregulating inflammatory cytokines (Wang et al., 2018). A recent study showed that EA stimulation at Baihui (GV20) and Zusanli (ST36) increased the abundance of anti-inflammatory probiotics such as Lactobacillus and Bifidobacterium (Xu et al., 2013), reduced the abundance of *E. coli* and *B. fragilis*, and restored the learning and memory of D-galactose-treated aging rats by inhibiting the level of lipopolysaccharide (He et al., 2021). Recent studies showed that acupuncture on GB34 and ST36 significantly reduced the amount of pro-inflammatory pathogenic bacteria such as Bacteroidetes and inhibited glial cell activation and apoptosis, thus reducing neuroinflammation (Jang et al., 2020). For example, EA reduced the abundance of pathogenic bacteria, such as Streptococcus (Wang et al., 2021). It was also found that moxibustion on ST25 and RN6 altered the intestinal flora composition by decreasing *E. coli* and *B. fragilis*, which are highly associated with colonic inflammation (Wang, 2012). Short-chain fatty acids (SCFAs), secreted by probiotics, can inhibit the colonization and growth of pathogenic bacteria to the intestinal mucosa and reduce the production of inflammatory cytokines, thus regulating the intestinal barrier and immune system. Studies demonstrated that 12-week acupuncture therapy increased the relative abundance of SCFAs and produced bacteria such as Lachnospira, Coprococcus, Roseburia, and *Roseburia faecis*, suggesting that acupuncture may help to restore the balance of intestinal microbiota. EA can also induce intestinal epithelial cells to secrete IL-18 and antimicrobial by stimulating SCFAs (Guillemot-Legris and Muccioli, 2017). After moxibustion at RN4 for 40 days, the intestinal SCFA content of elder rats increased to some extent, especially acetic acid, propionic acid, and isobutyric acid (Ouyang et al., 2022).

##### 3.2.1.2 The second way is modulating immune cells and the system

Multiple studies have revealed that preventing immune overreaction is one of the key principles of acupuncture and moxibustion therapy in attenuating inflammation in numerous diseases (Li N. et al., 2021). In a normal state, Th17 immune cells secrete pro-inflammatory cytokines that induce inflammation against pathogen infections (Geng and Xue, 2016); while on the other hand, Treg immune cells inhibit intestinal inflammation. However, when the body is stimulated, initial CD4+ T cells substantially differentiate into Th17 immune cells and disrupt the balance, which may lead to abnormal immune responses and intestinal inflammation. Studies showed that EA at Tianshu (ST25) and Zusanli (ST36) has the ability to restore the normal ratio of Treg and Th17 cells in spleen lymphocytes of UC mice (Sun et al., 2017), which helps to improve intestinal inflammatory response (Liu Z. et al., 2022). Furthermore, EA could also relieve intestinal mucosal inflammation and autoimmune diseases by regulating marker transcription factors of immune cells such as RORyt and Foxp, as well as their metabolic pathways (Liu Z. et al., 2022).

In addition, immune cells secrete both pro-inflammatory and anti-inflammatory cytokines. The former includes IL-17, IL-21, and IL-22, while the latter includes IL-10 and TGF-β. The dynamic balance between these two types of cytokines helps maintain the stability of the intestinal environment, while their imbalance may lead to abnormal immune responses and severe inflammatory reactions in IBD patients. Studies have shown that EA at ST36, LI11, and ST25 can upregulate IL-10 by increasing levels of CD3 + CD4+ T cells and Treg cells in the mesenteric lymph nodes and downregulate the levels of Th17 cells and TNF-α to suppress intestinal inflammation (Xie et al., 2020). Previous studies also demonstrated that moxibustion could inhibit chemotaxis and cell migration into inflamed tissues by promoting neutrophil apoptosis (Wang, 2012) and downregulating cytokines (Zhang et al., 2022), such as IL-1β, IL-17, IFN-γ, and TNF-α, to improve inflammatory status and memory impairments. Moreover, moxibustion could regulate the colonic immune response by inhibiting TNFα and IL12 expression in the colon tissues of UC rats (Qi et al., 2018). Additionally, EA stimulation in Aβ25-35-treated rats can downregulate IL-1β and TNF-α in the prefrontal cortex and hippocampus, attenuating inflammatory responses and improving memory impairment.

When the intestinal mucosa is damaged, the monocyte–macrophage system will be initiated. After that the initiation, the TLR4/MyD88/NF-κB signaling pathways mediated by toll-like receptors (TLRs) would be abnormally activated, resulting in the amplification of inflammatory responses (Shen et al., 2020). Studies have shown that manual acupuncture (MA) at ST36 could modulate the macrophage polarization balance in an inflamed site by inhibiting pro-inflammatory M1 macrophages and promoting anti-inflammatory M2 macrophages simultaneously. Additionally, after moxibustion at RN4, the related inflammatory factors of myeloid differentiation factor 88 (MyD88)/nuclear factor kappa-B (NF-κB) pathway in intestinal tissue were found significantly decreased in elder mice (Ouyang et al., 2022). One study (Dong et al., 2018) indicated that EA at ST36 and BL60 (Kunlun) could inhibit expression of TLR4, MYD88, and NF-kB, so as to improve inflammation reactions. Moreover, studies show that EA at GV20, Dazhui (GV14), and Shenshu (BL23) could reduce the expression of NLRP3 inflammasome and neuronal damage in the hippocampal CA1 area. In the irritable bowel syndrome (IBS) model, EA at ST36 has been found to decrease the level of mast cells (MC) and downregulate interleukin-1β (IL-1β) in colon tissue, thus improving visceral hypersensitivity (Wang et al., 2019). Acupuncture and moxibustion stimulation can also release large quantities of bio-active mediators such as histamine, substance P (SP), and 5-hydroxytryptamine (5-HT) from activated MC, which directly stimulate peripheral nerve receptors, resulting in a healing effect on colonic mucosal damage (Shi, 2011). Additionally, research suggests that EA at ST36 upregulates cadherin, claudin-1, and zonula occludens-1 (ZO-1) levels in colon tissue, which prevents granulocyte infiltration into damaged areas and reduces gastrointestinal inflammation (Liu G.-H. et al., 2020). Studies also showed that EA at ST36, ST37, ST39, and other acupoints could attenuate inflammation by inhibiting the activity of myeloperoxidase (MPO), which is produced by granulocytes and can activate neutrophils (Yang et al., 2020). Wu et al. demonstrated that acupuncture therapy could suppress oxidative stress in UC rats by regulating related markers such as SOD, CAT, and MDA, which are involved in the pathogenesis and progression of IBD (Liu Z. et al., 2022).

##### 3.2.1.3 The third way is to reduce inflammation through the neuroendocrine system

According to Chen et al. (2020) both acupuncture and moxibustion could reduce the persistent inflammatory state by downregulating the expression of sympathetic neuropeptides, such as neurokinin A (NKA), neurokinin B (NKB), SP, and vasoactive intestinal peptide (VIP). Additionally, acupuncture at ST36 stimulates the sympathetic nerve to release norepinephrine (NE), which specifically binds to the β2-adrenergic receptor (β2AR) and enables anti-inflammatory action, as reported by Xiao et al. Along with this study, the hypothalamic–pituitary–adrenal (HPA) axis, mainly composed of the hypothalamic PVN, adenohypophysis, and the adrenal cortex, also plays crucial roles. Studies indicate that acupuncture directly regulates the endogenous HPA axis, which prevents the inflammatory response mediated by exogenous cortisol overdose. In addition, the somatic afferents activated by acupuncture can be conveyed to the corresponding nuclei of the brainstem and hypothalamus (Li N. et al., 2021). Studies have also proven that moxibustion could ameliorate anxiety behavior in DSS-induced colitis, partially by improving the balance of the HPA axis (Wei et al., 2019). Meanwhile, Wei et al. revealed that acupuncture can promote adreno-cortico-tropic hormone (ACTH), shifting the hypo-responsiveness of the HPA axis to hyper-responsiveness, thus attenuating inflammation (Wei et al., 2017).

Recently, the anti-inflammatory effect of acupuncture, mediated by CAIP, has gained more and more attention worldwide. Studies show that EA promotes acetylcholine (ACh) release and activates α7 nicotinic acetylcholine receptors (α7nAChRs) in mesenteric adipose tissues (mWAT), both of which are the key components of CAIP, thus increasing the anti-inflammatory factor TGF-1β and IL10 expression (Jiang et al., 2019), as well as inhibiting pro-inflammatory TNF-α production. In AD model rats, auricular acupuncture (AA) was found to be involved in the synthesis of choline acetyltransferase (ChAT) in the hippocampus and the regulation of abnormal astrocytic hyperactivity (Miao et al., 2009). In addition, Ma et al. found that EA pretreatment at Baihui (GV 20) increases the expression of anti-inflammatory cytokines by conversion of microglia from the M1 to M2 phenotype through α7nAChR signaling (Ma et al., 2019). Then, the upregulated α7nAChRs would activate the JAK2/STAT3 pathway (downstream pathways) and reduce the expression of TNF-α and IL-6 in the hippocampus to protect neurons (Cao et al., 2021). Researches (Salazar et al., 2017) also show that EA at LI‐4 and LI‐11, and Du‐14 and Du‐20 mobilized mesenchymal stem cells (MSC) into the systemic circulation which could enhance tissue repair and increase anti‐inflammatory cytokine production. In addition, EA also mediates anti-inflammatory responses through the upregulation of five subtypes of muscarinic receptors (M1, M2, M3, M4, and M5) on cholinergic neurons in various areas of the brain.

#### 3.2.2 Correction of the neuroendocrine system disorders

It is widely recognized that acupuncture and moxibustion therapy has gained popularity in regulating the autonomic nervous system (ANS) by stimulating the parasympathetic pathway. While vagal activity has been implicated as a possible mediator of acupuncture therapy (Lim et al., 2016). Acupuncture at ST36 releases adenosine and histamine (Huang et al., 2018) and activates the expression of the pERK pathway to protect neuronal degeneration and improve various neuronal dysfunctions (Mosher and Wyss-Coray, 2014). Moreover, acupuncture signaling has been reported to show potent effects in alleviating intestinal inflammation and barrier dysfunction by triggering the vagal-adrenal medulla reflex and significantly increasing dopamine (DA) plasma levels (Li Y. et al., 2021). It has been confirmed that one of the mechanisms of acupuncture and moxibustion therapy is to downregulate the expression of pro-apoptotic protein Bax in the hippocampus, upregulate the expression of anti-apoptotic protein Bcl-2, and inhibit the initiation of endogenous apoptosis (Du et al., 2022). Scholars also found that EA at Baihui can increase the expression of neurotrophins such as brain-derived neurotrophic factor (BDNF) in the cerebral cortex so as to repair damaged cells and improve the antioxidant stress capacity of the body. Related clinical RCTs have pointed out that moxibustion could effectively alleviate cognitive dysfunction through regulating cognition- or aging-related gene expressions (Ji et al., 2021), brain functional connectivity, and synaptic plasticity signaling pathways, including p38 MAPKs, PI3K/AKT, and NF-kappa-B (Lai et al., 2019). In murine models for vascular dementia (VaD), it has been identified that EA could improve cognitive impairment by improving the proliferation of hippocampal neuro-stem cells (NSCs) to repair neurons and improve brain function (Ahn et al., 2016). In addition, studies have indicated that acupoint stimulation has the potential to protect hippocampal neurons by preventing apoptosis, scavenging toxic proteins, and inhibiting the production of amyloid β-peptide and the phosphorylation of Tau protein (Zhang et al., 2023).

## 4 How is the body’s metabolism and intestinal barrier function regulated?

### 4.1 Initiative regulation of endocrine metabolism mediated by brain-intestinal peptides

Brain-intestinal peptides are small molecules distributed in the brain and the gastrointestinal (GI) tract that transmit information in both directions in the GBA, mainly including SP, VIP, cholecystokinin, nitric oxide (NO), and 5-HT. These small molecules secreted by endocrine cells of the gut are involved in the maintenance of the stability of GI function at both central and peripheral levels by regulating the expression of hormones and neurotransmitters. GBA dysfunctions would affect the secretion of brain–gut peptides, which may further lead to certain neuro-degenerative diseases and gastrointestinal dysfunction. There is considerable evidence that EA could restore the GBA in certain intestinal disorders to balanced levels so as to regulate internal environmental stability. Experimentally, it was demonstrated that increased 5-HT and calcitonin gene-related peptide (CGRP) levels and decreased neuropeptide Y (NPY) content were found in the rat model of diarrheal irritable bowel syndrome (D-IBS) (Sun et al., 2015). However, EA attenuated the above changes of 5-HT, CGRP, and NPY in GBA to a normal state (Sun et al., 2015). Among them, 5-HT is a major transmitter of the GBA, the reduction of which, together with CGRP, has been shown to attenuate visceral sensitivity. Similarly, Yu et al. reported that EA at CV12, RN6, ST36, and LR3 effectively alleviates neuronal loss and apoptosis by regulating brain–gut peptides, such as NPY, cholecystokinin, somatostatin, gastrin, and peptide YY (Yu et al., 2020). Glial cells constitute almost half of the cells in the central nervous system (CNS), which have been highlighted in many aspects of CNS function. Studies suggest that EA at ST36 significantly increased the expression of astrocytic and microglial (OX-42) marker cells in the dorsal vagal complex (DVC) and regulated gastrointestinal activity (Yu, 2020). There is a study by Bao et al. (2022a,b) indicating that metabolic changes and behavior in the central nervous system may be influenced by moxibustion through the kynurenine pathway (KP) along the gut–brain axis.

### 4.2 Regulation of energetic metabolism and improvement of insulin resistance

First of all, consistent with the theory of innervation of neural segments, needling adjacent acupoints helps to reduce blood glucose and protect the shape of islets (Feng et al., 2018). The same results were also found in the study of Xu et al. (2017), which pointed out that EA restored serum β-glucose and inositol in rats with chronic atrophic gastritis by normalizing a number of CAG-induced metabolomics changes. In addition, studies have shown that acupuncture reduces the insulin resistance of model rats by restoring the level of insulin signaling-related molecules such as IRS-1, IRS-2, Akt2, and GLUT4 to normal, activating the signal transduction pathway of the phosphatidylinositol 3-kinase (PI3K)/Akt (Huang et al., 2016). For example (Liu et al., 2017) found that EA could increase the expression of GLUT1 and GLUT3 in the hippocampus and cortex so as to improve the learning and memory of APP/PS1 mice by regulating glucose metabolism. Another study suggests that EA may be considered as a new insulin sensitizer, which could improve insulin sensitivity and T2DM through various mechanisms, such as weight loss, anti-inflammatory, and the improvement of lipid metabolism and adipokines as well (Firouzjaei et al., 2016). EA at ST36 and BL23 is reported to reduce the level of fasting insulin (FINS) and protect islet B cell morphology so as to improve insulin sensitivity by increasing the expression of GLUT2 and GCK, both of which function together as “glucose sensor” (Feng et al., 2018) and reduce neuronal damage by promoting glucose metabolism. Moreover, studies also indicated that short-term AA could improve antioxidant capacity in people at high risk for diabetes, thus reducing the risk for dementia (Liu et al., 2008).

### 4.3 Improvement of gut barrier function

The gastrointestinal (GI) epithelial barrier is crucial for maintaining gut environmental homeostasis. Its integrity depends on the major nervous systems, enteric neuron function, intestinal permeability, tight junctions, and epithelial cell integrity (Song et al., 2019). Disruption of the intestinal mucosal barrier and increased intestinal permeability can lead to impaired nutrient and toxin absorption, causing the loss of neurons, astrocytes, microglia, and endothelial cells, which increases the risk of developing progressive cognitive impairment or dementia. It can be observed that EA at Sibai (ST2), Liangmen (ST21), and ST36 acupoints of CAG rats rendered the gastric mucosal folds and thickness of gastric mucosa (Xu et al., 2017). Moreover, moxibustion could restore the colonic mucosa to the level of healthy rats and decrease submucosal inflammatory cell infiltration and congestion in UC rats (Qi et al., 2018). Studies suggest that acupuncture at ST36 may regulate the integrity of the intestinal barrier by inducing the intestinal epithelial cells through the cholinergic pathway (Xie et al., 2020) to upregulate the expression of tight junctions and reduce apoptosis of intestinal epithelial cells (Guillemot-Legris and Muccioli, 2017). Furthermore, EA at ST36 can also attenuate the distortion of enteric glial cells, maintain intestinal barrier function, and reduce intestinal permeability, preventing toxins from trans-locating from the lumen into the blood.

## 5 Summary

Based on the analysis of several systematic reviews and randomized control trials (RCTs), we have identified the most commonly used acupuncture points to improve cognitive impairment through the MGB axis and their probable mechanisms in Supplementary Table 2. Out of all the relevant publications, Zusanli (ST36) is the most frequently used acupuncture point, followed by Tianshu (ST25). Acupuncture points on the spleen, stomach, and intestinal meridians accounted for nearly half of the frequency, consistent with the role of acupuncture in improving the internal environment of the stomach and intestines. Acupuncture points on the extremities are used more often than those on the abdomen or craniofacial region. Additionally, it is critical to ensure that patients experience the deqi sensation (Si et al., 2021), which is a sensation of soreness, distention, numbness, or heaviness. Clinical studies favored combinations of multiple acupoints, while single-acupoint treatment was more common in animal tests, usually using Baihui (GV 20) and Zusanli (ST36). Studies showed that single-site treatment could also exert multiple dimensions of therapeutic effects through different mechanisms, while most RCTs did not mention any side effects. This is an advantage of acupuncture over Western medicine, as it is multifaceted, multi-targeted, and safer.

With regard to the difference between the effects of EA and MA on cognitive impairment, it is generally recognized that EA stimulation shows a more beneficial effect on neurodegenerative diseases (Lin et al., 2018). Studies found that EA activated more callback brain regions and functional connectivity than MA (Liu et al., 2023) due to the electrical twitching of surrounding tissues it produced (Yamamoto et al., 2011). However, some studies (Mitchell and Shiri-Feshki, 2008) instead found that patients who received MA displayed a significant improvement in cognitive function compared to those who received EA. We could not make a conclusion about whether one treatment has a better cognitive improvement effect than the other due to the lack of studies of large sample sizes and high-quality evidence-based medical evidence. The probable reasons for the ambiguity are listed as follows. First, MA and EA might exert different therapeutic effects through respective mechanisms related to the characteristics of diseases (Yu et al., 2014). For example, (Lin et al., 2018) pointed out that EA might promote neuroprotection and neuronal regeneration through TrkB and NF-kB signal pathways, while MA could activate the BDNF and its downstream signaling pathways (Ferrón Sacri et al., 2009). Second, the effect of MA depends on acupuncture points, stimulating intensity (Jiang et al., 2013) and courses of treatment, as well as the “De-Qi” feeling (Lin et al., 2018). However, most of the study subjects are mice and their feedback on acupuncture could not be tested, which might affect the efficacy to some extent.

In 1990, the World Health Organization recognized auricular acupuncture (AA) as a microacupuncture system, which has a positive impact on regulating whole-body function. Studies found that AA could prevent or improve cognitive impairment by activating the “ear-vagus nerve reflex” (Rong et al., 2015) and regulating the expression of beta-amyloid protein in the brain. Recent studies indicated that AA could induce central anti-inflammatory reaction (Corsi-Zuelli et al., 2017), mainly via CAP (Liu C.-H. et al., 2020), and affect the HPA axis and cortisol levels as well (Kim et al., 2012). Moreover, AA could also modulate immune activation in the gut wall (Greenwood and Davison, 1987) and decrease intestinal permeability, thus likely modulating microbiota composition (Breit et al., 2018). Then, AA may further regulate the neuroimmune system, the neuroendocrine system, and brain regions associated with cognition (Liu C.-H. et al., 2020).

## 6 Conclusion and future directions

In summary, the MGB axis plays a significant role in the development of cognitive impairment. Several promising targets for improving cognitive impairment have been identified, including the species and diversity of intestinal flora, immune-inflammatory reactions, neural pathways, metabolism, and intestinal barrier. The acupuncture and moxibustion therapy has been proven to improve cognitive function mainly through regulating the neuroendocrine system to improve intestinal inflammation, suppressing excessive immune response through neurofeedback pathways, maintaining intestinal microecological stability, and improving the intestinal barrier.

This study reviews the possible ways in which acupuncture and moxibustion therapy modulates the MGB axis to improve cognitive impairment and analyzes the acupuncture points of high frequency. Exploring the relationship between acupuncture and moxibustion treatment and the central nervous system through the MGB axis helps to provide theoretical mechanisms for treatments that target the axis. However, more systematic analyses of the effects of the acupuncture and moxibustion treatment on specific microbiota species are still needed. Acupuncture and moxibustion treatment targeting the gastrointestinal tract, not just brain function, deserves more high-quality clinical studies, which may be an important future challenge for standardized clinical application. One study (Dong et al., 2018) indicated that EA at ST36 and BL60(Kunlun) could inhibit expression of TLR4, MYD88, and NF-kB, so as to improve inflammation reactions.

## Author contributions

JS: Visualization, Writing – original draft. XZ: Writing – original draft. JC: Writing – review & editing. RS: Conceptualization, Writing – review & editing. HC: Supervision, Writing – review & editing. HW: Supervision, Writing – review & editing.

## Glossary

MGB axis: microbiome–gut–brain axis
CNS: central nervous system
CI: cognitive impairment
EA: electroacupuncture
ANS: autonomic nervous system
BBB: blood–brain barrier
GBA: gut–brain axis
MIDs: metabolic inflammatory diseases
GF: germ-free
EECs: enteroendocrine cells
NSCs: neuro-stem cells
VaD: vascular dementia
SCFAs: short-chain fatty acids
ChAT: cholinergic-related enzyme acetylcholine transferase
α7nAChR: α7-nicotinic acetylcholine receptor
TLRs: toll-like receptors
MyD88: myeloid differentiation factor 88
NF-κB: nuclear factor kappa-B
IBS: irritable bowel syndrome
MC: mast cells
SP: substance P
HPA axis: hypothalamic–pituitary–adrenal axis

## References

[ref1] Ahn S. M. Kim Y. R. Kim H. N. Shin Y.-I. Shin H. K. Choi B. T. (2016). Electroacupuncture ameliorates memory impairments by enhancing oligodendrocyte regeneration in a mouse model of prolonged cerebral Hypoperfusion. Sci. Rep. 6:28646. doi: 10.1038/srep28646, PMID: 27350403 PMC4923909

[ref2] Arneth B. M. (2018). Gut–brain Axis biochemical Signalling from the gastrointestinal tract to the central nervous system: gut Dysbiosis and altered brain function. Postgrad. Med. J. 94, 446–452. doi: 10.1136/postgradmedj-2017-135424, PMID: 30026389

[ref3] Bao C. Huang J. Huangan W. Ma Y. Zhou H. Chen L.. (2022a). Moxibustion alleviates depression-like behavior in rats with Crohn’s disease by inhibiting the kynurenine pathway metabolism in the gut-brain Axis. Front. Neurosci. 16:1019590. doi: 10.3389/fnins.2022.1019590, PMID: 36570839 PMC9768219

[ref4] Bao C. Wu L. Wang D. Chen L. Jin X. Shi Y.. (2022b). Acupuncture improves the symptoms, intestinal microbiota, and inflammation of patients with mild to moderate Crohn’s disease: a randomized controlled trial. eClinicalMedicine 45:101300. doi: 10.1016/j.eclinm.2022.101300, PMID: 35198926 PMC8850329

[ref5] Bioque M. González-Rodríguez A. Garcia-Rizo C. Cobo J. Monreal J. A. Usall J.. (2021). Targeting the microbiome-gut-brain Axis for improving cognition in schizophrenia and major mood disorders: a narrative review. Prog. Neuro-Psychopharmacol. Biol. Psychiatry 105:110130. doi: 10.1016/j.pnpbp.2020.110130, PMID: 33045322

[ref6] Breit S. Kupferberg A. Rogler G. Hasler G. (2018). Vagus Nerve as Modulator of the Brain–Gut Axis in Psychiatric and Inflammatory Disorders. Front. Psych. 9:44. doi: 10.3389/fpsyt.2018.00044, PMID: 29593576 PMC5859128

[ref7] Camkurt M. A. Lavagnino L. Zhang X. Y. Teixeira A. L. (2018). Liraglutide for psychiatric disorders: clinical evidence and challenges. Horm. Mol. Biol. Clin. Invest. 36. doi: 10.1515/hmbci-2018-0031, PMID: 30020885

[ref8] Cao Y. Wang L. Lin L.-T. Wang X.-R. Ma S.-M. Yang N.-N.. (2021). Acupuncture attenuates cognitive deficits through Α7nAChR mediated anti-inflammatory pathway in chronic cerebral Hypoperfusion rats. Life Sci. 266:118732. doi: 10.1016/j.lfs.2020.118732, PMID: 33160996

[ref9] Carabotti M. Scirocco A. Maselli M. A. Severi C. (2015). The gut-brain Axis: interactions between enteric microbiota, central and enteric nervous systems. Ann. Gastroenterol. 28, 203–209. 25830558 PMC4367209

[ref10] Chang C.-H. Lin C.-H. Lane H.-Y. (2020). D-glutamate and gut microbiota in Alzheimer’s disease. Int. J. Mol. Sci. 21:2676. doi: 10.3390/ijms21082676, PMID: 32290475 PMC7215955

[ref9001] Chen Y. Gao Y. Lu W. Gao W. (2020). Influence of acupuncture on the expression of VIP, SP, NKA and NKB, cAMP/cGMP and HE content and treatment of bronchial asthma in rats. Cell. Mol. Biol. 66, 29–35. 33040809

[ref11] Corsi-Zuelli F. M. Graças D. Brognara F. Quirino G. F. D. S. Hiroki C. H. Fais R. S.. (2017). Neuroimmune interactions in schizophrenia: focus on Vagus nerve stimulation and activation of the Alpha-7 nicotinic acetylcholine receptor. Front. Immunol. 8:618. doi: 10.3389/fimmu.2017.00618, PMID: 28620379 PMC5449450

[ref12] Cui J. Song W. Jin Y. Huihao X. Fan K. Lin D.. (2021). Research Progress on the mechanism of the acupuncture regulating neuro-endocrine-immune network system. Vet. Sci. 8:149. doi: 10.3390/vetsci8080149, PMID: 34437474 PMC8402722

[ref13] Cussotto S. Sandhu K. V. Dinan T. G. Cryan J. F. (2018). The neuroendocrinology of the microbiota-gut-brain Axis: a Behavioural perspective. Front. Neuroendocrinol. 51, 80–101. doi: 10.1016/j.yfrne.2018.04.002 29753796

[ref14] Dong Z.-Q. Zhu J. De-zhao L. Chen Q. Ying-ling X. (2018). Effect of Electroacupuncture in ‘Zusanli’ and ‘Kunlun’ Acupoints on TLR4 signaling pathway of adjuvant arthritis rats. Am. J. Ther. 25, e314–e319. doi: 10.1097/MJT.0000000000000477, PMID: 27574922

[ref15] Du K. Yang S. Wang J. Zhu G. (2022). Acupuncture Interventions for Alzheimer’s Disease and Vascular Cognitive Disorders: A Review of Mechanisms. Oxidative Med. Cell. Longev. 2022, 1–18. doi: 10.1155/2022/6080282, PMID: 36211826 PMC9534683

[ref16] Erny D. Hrabě A. L. de Angelis D. Jaitin P. W. Staszewski O. David E.. (2015). Host microbiota constantly control maturation and function of microglia in the CNS. Nat. Neurosci. 18, 965–977. doi: 10.1038/nn.4030, PMID: 26030851 PMC5528863

[ref17] Feng Y. Fang Y. Wang Y. Hao Y. (2018). Acupoint therapy on diabetes mellitus and its common chronic complications: a review of its mechanisms. Biomed. Res. Int. 2018, 1–9. doi: 10.1155/2018/3128378, PMID: 30426006 PMC6217896

[ref18] Ferrón Sacri R. Angeles M.-T. M. Helena M. Ignacio F. Kerrie T. Blasco María A.. (2009). Telomere shortening in neural stem cells disrupts neuronal differentiation and neuritogenesis. J. Neurosci. 29, 14394–14407. doi: 10.1523/JNEUROSCI.3836-09.2009, PMID: 19923274 PMC6665809

[ref19] Firouzjaei A. Li G.-C. Wang N. Liu W.-X. Zhu B.-M. (2016). Comparative evaluation of the therapeutic effect of metformin monotherapy with metformin and acupuncture combined therapy on weight loss and insulin sensitivity in diabetic patients. Nutr. Diabetes 6:e209. doi: 10.1038/nutd.2016.16, PMID: 27136447 PMC4895377

[ref20] Fox M. Knorr D. A. Haptonstall K. M. (2019). Alzheimer’s disease and symbiotic microbiota: An evolutionary medicine perspective. Ann. N. Y. Acad. Sci. 1449, 3–24. doi: 10.1111/nyas.14129 31180143 PMC9495288

[ref21] Geng X. Xue J. (2016). Expression of Treg/Th17 cells as well as related cytokines in patients with inflammatory bowel disease. Pak. J. Med. Sci. 32, 1164–1168. doi: 10.12669/pjms.325.10902, PMID: 27882014 PMC5103126

[ref22] Greenwood B. Davison J. S. (1987). The relationship between gastrointestinal motility and secretion. Am. J. Physiol. 252, G1–G7. doi: 10.1152/ajpgi.1987.252.1.G1 3544864

[ref23] Guillemot-Legris O. Muccioli G. G. (2017). Obesity-induced Neuroinflammation: beyond the hypothalamus. Trends Neurosci. 40, 237–253. doi: 10.1016/j.tins.2017.02.005, PMID: 28318543

[ref24] He C. Huang Z.-S. Chao-Chao Y. Wang X.-S. Jiang T. Miao W.. (2021). Preventive Electroacupuncture ameliorates D-galactose-induced Alzheimer’s disease-like inflammation and memory deficits, probably via modulating the microbiota–gut–brain Axis. Iran. J. Basic Med. Sci. 24, 341–348. doi: 10.22038/ijbms.2021.49147.11256, PMID: 33995945 PMC8087854

[ref25] Huang J. Shen M. Qin X. Manli W. Liang S. Huang Y. (2020). Acupuncture for the treatment of Alzheimer’s disease: An overview of systematic reviews. Front. Aging Neurosci. 12:574023. doi: 10.3389/fnagi.2020.574023, PMID: 33328956 PMC7729156

[ref26] Huang M. Wang X. Xing B. Yang H. Sa Z. Zhang D.. (2018). Critical roles of TRPV2 channels, histamine H1 and adenosine A1 receptors in the initiation of Acupoint signals for acupuncture analgesia. Sci. Rep. 8:6523. doi: 10.1038/s41598-018-24654-y, PMID: 29695862 PMC5916903

[ref27] Huang X.-Y. Zhang L. Sun J. Neng-Gui X. Yi W. (2016). Acupuncture alters expression of insulin signaling related molecules and improves insulin resistance in OLETF rats. Evid. Based Complement. Alternat. Med. 2016, 1–7. doi: 10.1155/2016/9651592, PMID: 27738449 PMC5055976

[ref28] Jang J.-H. Yeom M.-J. Ahn S. Ju-Young O. Ji S. Kim T.-H.. (2020). Acupuncture inhibits Neuroinflammation and gut microbial Dysbiosis in a mouse model of Parkinson’s disease. Brain Behav. Immun. 89, 641–655. doi: 10.1016/j.bbi.2020.08.015, PMID: 32827699

[ref29] Ji S. Duan J. Hou X. Zhou L. Qin W. Niu H.. (2021). The role of acupuncture improving cognitive deficits due to Alzheimer’s disease or vascular diseases through regulating neuroplasticity. Neural Plast. 2021, 1–16. doi: 10.1155/2021/8868447, PMID: 33505460 PMC7815402

[ref30] Jiang T. Meiyan W. Zhang Z. Yan C. Ma Z. He S.. (2019). Electroacupuncture attenuated cerebral ischemic injury and Neuroinflammation through Α7nAChR-mediated inhibition of NLRP3 Inflammasome in stroke rats. Mol. Med. 25:22. doi: 10.1186/s10020-019-0091-4, PMID: 31117961 PMC6530013

[ref31] Jiang Y. Wang H. Liu Z. Dong Y. Dong Y. Xiang X.. (2013). Manipulation of and sustained effects on the human brain induced by different modalities of acupuncture: An fMRI study. PLoS One 8:e66815. doi: 10.1371/journal.pone.0066815, PMID: 23840533 PMC3696086

[ref32] Kesika P. Suganthy N. Sivamaruthi B. S. Chaiyasut C. (2021). Role of gut-brain Axis, gut microbial composition, and probiotic intervention in Alzheimer’s disease. Life Sci. 264:118627. doi: 10.1016/j.lfs.2020.118627, PMID: 33169684

[ref33] Khodabakhsh P. Bazrgar M. Dargahi L. Mohagheghi F. Taei A. A. Parvardeh S.. (2021). Does Alzheimer’s disease stem in the gastrointestinal system? Life Sci. 287:120088. doi: 10.1016/j.lfs.2021.120088, PMID: 34715145

[ref34] Kim H. Chen L. Lim G. Sung B. Wang S. McCabe M. F.. (2012). Brain Indoleamine 2,3-dioxygenase contributes to the comorbidity of pain and depression. J. Clin. Investig. 122, 2940–2954. doi: 10.1172/JCI61884, PMID: 22751107 PMC3408737

[ref35] Lach G. Schellekens H. Dinan T. G. Cryan J. F. (2018). Anxiety, depression, and the microbiome: a role for gut peptides. Neurotherapeutics 15, 36–59. doi: 10.1007/s13311-017-0585-0, PMID: 29134359 PMC5794698

[ref36] Lai H.-C. Chang Q.-Y. Hsieh C.-L. (2019). Signal transduction pathways of acupuncture for treating some nervous system diseases. Evid. Based Complement. Alternat. Med. 2019, 1–37. doi: 10.1155/2019/2909632, PMID: 31379957 PMC6657648

[ref39] Lim H.-D. Kim M.-H. Lee C.-Y. Namgung U. (2016). Anti-inflammatory effects of acupuncture stimulation via the Vagus nerve. PLoS One 11:e0151882. doi: 10.1371/journal.pone.0151882, PMID: 26991319 PMC4798687

[ref9002] Lin C. J. Yeh M. L. Wu S. F. Chung Y. C. Lee J. C. (2022). Acupuncture-related treatments improve cognitive and physical functions in Alzheimer’s disease: A systematic review and meta-analysis of randomized controlled trials. Clin. Rehabil. 36, 609–635. doi: 10.1177/02692155221079117 35229686

[ref40] Lin D. Zhang J. Zhuang W. Yan X. Yang X. Lin S.. (2018). The effect of Electroacupuncture versus manual acupuncture through the expression of TrkB/NF- κ B in the subgranular zone of the dentate gyrus of telomerase-deficient mice. Evid. Based Complement. Alternat. Med. 2018, 1–11. doi: 10.1155/2018/1013978, PMID: 29849690 PMC5937603

[ref37] Li N. Guo Y. Gong Y. Zhang Y. Fan W. Yao K.. (2021). The anti-inflammatory actions and mechanisms of acupuncture from Acupoint to target organs via neuro-immune regulation. J. Inflamm. Res. 14, 7191–7224. doi: 10.2147/JIR.S341581, PMID: 34992414 PMC8710088

[ref45] Liu C. F. Yu L. F. Lin C. H. Lin S. C. (2008). Effect of auricular pellet acupressure on antioxidative systems in high-risk diabetes mellitus. J. Altern. Complement. Med. 14, 303–307. doi: 10.1089/acm.2006.6064, PMID: 18399759

[ref44] Liu C.-H. Yang M.-H. Zhang G.-Z. Wang X.-X. Li B. Li M.. (2020). Neural networks and the anti-inflammatory effect of transcutaneous auricular Vagus nerve stimulation in depression. J. Neuroinflammation 17:54. doi: 10.1186/s12974-020-01732-5, PMID: 32050990 PMC7017619

[ref43] Liu G.-H. Liu H.-M. Chen Y.-S. Lee T.-Y. (2020). Effect of Electroacupuncture in mice with dextran sulfate sodium-induced colitis and the influence of gut microbiota. Evid. Based Complement. Alternat. Med. 2020, 1–13. doi: 10.1155/2020/2087903, PMID: 32419794 PMC7204379

[ref46] Liu G.-H. Zhuo X.-C. Huang Y.-H. Liu H.-M. Ren-Chin W. Kuo C.-J.. (2022). Alterations in gut microbiota and upregulations of VPAC2 and intestinal tight junctions correlate with anti-inflammatory effects of Electroacupuncture in colitis mice with sleep fragmentation. Biology 11:962. doi: 10.3390/biology11070962, PMID: 36101343 PMC9311573

[ref42] Liu J.-p. Li Y.-y. Yang K.-z. Shu-feng Shi Y. Gong Z. T. Tong Y.. (2023). Electroacupuncture and manual acupuncture at LR3 and ST36 have attenuating effects on hypertension and subsequent cognitive dysfunction in spontaneously hypertensive rats: a preliminary resting-state functional magnetic resonance imaging study. Front. Neurosci. 17:1129688. doi: 10.3389/fnins.2023.1129688, PMID: 36968479 PMC10033598

[ref9003] Liu W. Zhuo P. Li L. Jin H. Lin B. Zhang Y., (2017). Activation of brain glucose metabolism ameliorating cognitive impairment in APP/PS1 transgenic mice by electroacupuncture. Free radical biology & medicine, 112, 174–190. doi: 10.1016/j.freeradbiomed.2017.07.024 28756309

[ref41] Liu Z. Jiao Y. Yu T. Wang H. Zhang Y. Liu D.. (2022). A review on the immunomodulatory mechanism of acupuncture in the treatment of inflammatory bowel disease. Evid. Based Complement. Alternat. Med. 2022, 1–9. doi: 10.1155/2022/8528938, PMID: 35075366 PMC8783701

[ref38] Li Y. Guochen X. Sen H. Hong W. Dai Y. Zhang W.. (2021). Electroacupuncture alleviates intestinal inflammation and barrier dysfunction by activating dopamine in a rat model of intestinal Ischaemia. Acupunct. Med. 39, 208–216. doi: 10.1177/0964528420922232, PMID: 32517478

[ref47] Ma Z. Zhang Z. Bai F. Jiang T. Yan C. Wang Q. (2019). Electroacupuncture pretreatment alleviates cerebral ischemic injury through Α7 nicotinic acetylcholine receptor-mediated phenotypic conversion of microglia. Front. Cell. Neurosci. 13:537. doi: 10.3389/fncel.2019.00537, PMID: 31866829 PMC6908971

[ref48] Miao T. Jiang T. S. Dong Y. H. Jiang N. C. (2009). Effects of auricular acupuncture on the memory and the expression of ChAT and GFAP in model rats with Alzheimer's disease. Zhongguo Zhen Jiu 29, 827–832. 19873921

[ref49] Mitchell A. J. Shiri-Feshki M. (2008). Temporal trends in the Long term risk of progression of mild cognitive impairment: a pooled analysis. J. Neurol. Neurosurg. Psychiatry 79, 1386–1391. doi: 10.1136/jnnp.2007.142679, PMID: 19010949

[ref50] Mosher K. I. Wyss-Coray T. (2014). Microglial dysfunction in brain aging and Alzheimer’s disease. Biochem. Pharmacol. 88, 594–604. doi: 10.1016/j.bcp.2014.01.008, PMID: 24445162 PMC3972294

[ref51] Ouyang X. Duan H. Jin Q. Luo X. Han L. Zhao B.. (2022). Moxibustion may delay the aging process of Wistar rats by regulating intestinal microbiota. Biomed. Pharmacother. 146:112147. doi: 10.1016/j.biopha.2021.112147, PMID: 34810050

[ref52] Poole S. Singhrao S. K. Kesavalu L. Curtis M. A. Crean S. J. (2013). Determining the presence of Periodontopathic virulence factors in short-term postmortem Alzheimer’s disease brain tissue. J. Alzheimers Dis. 36, 665–677. doi: 10.3233/JAD-121918, PMID: 23666172

[ref53] Poutahidis T. Kearney S. M. Levkovich T. Qi P. Varian B. J. Lakritz J. R.. (2013). Microbial symbionts accelerate wound healing via the neuropeptide hormone oxytocin. PLoS One 8:e78898. doi: 10.1371/journal.pone.0078898, PMID: 24205344 PMC3813596

[ref54] Pratchett T. (2015). A global assessment of dementia, now and in the future. Lancet Neurol. 386:931. doi: 10.1016/S0140-6736(15)00117-8, PMID: 26369447

[ref55] Qi Q. Liu Y.-N. Jin X.-M. Zhang L.-S. Wang C. Bao C.-H.. (2018). Moxibustion treatment modulates the gut microbiota and immune function in a dextran Sulphate sodium-induced colitis rat model. World J. Gastroenterol. 24, 3130–3144. doi: 10.3748/wjg.v24.i28.3130, PMID: 30065559 PMC6064969

[ref56] Rong P.-J. Zhao J.-J. Li Y.-Q. Litscher D. Li S.-y. Ingrid Gaischek X.. (2015). Auricular acupuncture and biomedical research—a promising Sino-Austrian research cooperation. Chin. J. Integr. Med. 21, 887–894. doi: 10.1007/s11655-015-2090-9, PMID: 26631173

[ref57] Rooks M. G. Garrett W. S. (2016). Gut microbiota, metabolites and host immunity. Nat. Rev. Immunol. 16, 341–352. doi: 10.1038/nri.2016.42, PMID: 27231050 PMC5541232

[ref58] Salazar T. E. Richardson M. R. Beli E. Ripsch M. S. George J. Kim Y.. (2017). Electroacupuncture promotes central nervous system-dependent release of mesenchymal stem cells. Stem Cells 35, 1303–1315. doi: 10.1002/stem.2613 28299842 PMC5530374

[ref59] Sampson T. R. Debelius J. W. Thron T. Janssen S. Shastri G. G. Ilhan Z. E.. (2016). Gut microbiota regulate motor deficits and Neuroinflammation in a model of Parkinson’s disease. Cells 167, 1469–1480.e12. doi: 10.1016/j.cell.2016.11.018, PMID: 27912057 PMC5718049

[ref60] Severance E. G. Gressitt K. L. Stallings C. R. Origoni A. E. Sunil Khushalani F. Leweke M.. (2013). Discordant patterns of bacterial translocation markers and implications for innate immune imbalances in schizophrenia. Schizophr. Res. 148, 130–137. doi: 10.1016/j.schres.2013.05.018, PMID: 23746484 PMC3732507

[ref61] Shen N. Wang Z. Wang C. Zhang J. Liu C. (2020). Methane alleviates inflammation and apoptosis of dextran sulfate sodium-induced inflammatory bowel diseases by inhibiting toll-like receptor 4 (TLR4)/myeloid differentiation factor 88 (MyD88)/nuclear translocation of nuclear factor-ΚB (NF-ΚB) and endoplasmic reticulum stress pathways in mice. Med. Sci. Monit. 26:e922248. doi: 10.12659/MSM.922248 32500859 PMC7297035

[ref62] Shi Y. (2011). Moxibustion activates mast cell degranulation at the ST25 in rats with colitis. World J. Gastroenterol. 17, 3733–3738. doi: 10.3748/wjg.v17.i32.3733, PMID: 21990955 PMC3181459

[ref63] Si X. Han S. Zhang K. Zhang L. Sun Y. Jiayue Y.. (2021). The temporal dynamics of EEG microstate reveals the neuromodulation effect of acupuncture with Deqi. Front. Neurosci. 15:715512. doi: 10.3389/fnins.2021.715512, PMID: 34720853 PMC8549605

[ref64] Song G. Fiocchi C. Achkar J.-P. (2019). Acupuncture in inflammatory bowel disease. Inflamm. Bowel Dis. 25, 1129–1139. doi: 10.1093/ibd/izy371 30535303

[ref66] Sun J. Xiaoliang W. Meng Y. Cheng J. Ning H. Peng Y.. (2015). Electro-acupuncture decreases 5-HT, CGRP and increases NPY in the brain-gut Axis in two rat models of diarrhea-predominant irritable bowel syndrome(D-IBS). BMC Complement. Altern. Med. 15:340. doi: 10.1186/s12906-015-0863-5, PMID: 26419631 PMC4589130

[ref67] Sun J. Zhang H. Wang C. Yang M. Shyang Chang Y. Geng H. Y.. (2017). Regulating the balance of Th17/Treg via Electroacupuncture and Moxibustion: An ulcerative colitis mice model based study. Evid. Based Complement. Alternat. Med. 2017, 1–13. doi: 10.1155/2017/7296353, PMID: 29391874 PMC5748152

[ref65] Su X.-T. Wang L.-Q. Li J.-L. Na Zhang L. Wang G.-X. S. Yang J.-W.. (2020). Acupuncture therapy for cognitive impairment: a Delphi expert consensus survey. Front. Aging Neurosci. 12:596081. doi: 10.3389/fnagi.2020.596081, PMID: 33328975 PMC7732673

[ref68] Tooley K. L. (2020). Effects of the human gut microbiota on cognitive performance, brain structure and function: a narrative review. Nutrients 12:3009. doi: 10.3390/nu12103009, PMID: 33007941 PMC7601389

[ref73] Wang J. Xiao L. D. Wang K. Luo Y. Li X. (2020). "cognitive impairment and associated factors in rural elderly in North China". J. Alzheimers Dis. 77, 1241–1253. doi: 10.3233/JAD-200404, PMID: 32925043

[ref70] Wang L. An J. Song S. Mei M. Li W. Ding F.. (2020). Electroacupuncture preserves intestinal barrier integrity through modulating the gut microbiota in DSS-induced chronic colitis. Life Sci. 261:118473. doi: 10.1016/j.lfs.2020.118473, PMID: 32971101

[ref71] Wang M. Chen Y. Wang Y. Li Y. Zheng H. Ma F.. (2018). The effect of probiotics and polysaccharides on the gut microbiota composition and function of weaned rats. Food Funct. 9, 1864–1877. doi: 10.1039/C7FO01507K, PMID: 29521393

[ref72] Wang T.-Q. Li L.-R. Tan C.-X. Yang J.-W. Shi G.-X. Wang L.-Q.. (2021). Effect of Electroacupuncture on gut microbiota in participants with knee osteoarthritis. Front. Cell. Infect. Microbiol. 11:597431. doi: 10.3389/fcimb.2021.597431, PMID: 34671567 PMC8521167

[ref69] Wang X.-M. (2012). Moxibustion inhibits Interleukin-12 and tumor necrosis factor alpha and modulates intestinal Flora in rat with ulcerative colitis. World J. Gastroenterol. 18, 6819–6828. doi: 10.3748/wjg.v18.i46.6819, PMID: 23239920 PMC3520171

[ref74] Wang Y. L. Yang Shuai S. He W. Jing X. H. (2019). Electroacupuncture relieved visceral and referred Hindpaw hypersensitivity in colitis rats by inhibiting tyrosine hydroxylase expression in the sixth lumbar dorsal root ganglia. Neuropeptides 77:101957. doi: 10.1016/j.npep.2019.101957, PMID: 31400959

[ref76] Wei D. Xie L. Zhuang Z. Zhao N. Huang B. Tang Y.. (2019). Gut microbiota: a new strategy to study the mechanism of Electroacupuncture and Moxibustion in treating ulcerative colitis. Evid. Based Complement. Alternat. Med. 2019, 1–16. doi: 10.1155/2019/9730176, PMID: 31354859 PMC6632505

[ref75] Wei Y. Dong M. Zhong L. Liu J. Luo Q. Lv Y.. (2017). Regulation of hypothalamic-pituitary-adrenal Axis activity and immunologic function contributed to the anti-inflammatory effect of acupuncture in the OVA-induced murine asthma model. Neurosci. Lett. 636, 177–183. doi: 10.1016/j.neulet.2016.11.001 27816549

[ref77] Wennberg A. M. V. Gustafson D. Hagen C. E. Roberts R. O. Knopman D. Jack C.. (2016). Serum adiponectin levels, neuroimaging, and cognition in the Mayo Clinic study of aging. J. Alzheimer’s Dis. 53, 573–581. doi: 10.3233/JAD-151201, PMID: 27163809 PMC4955718

[ref78] World Health Organization (1991). Report on the working group on auricular acupuncture nomenclature, Lyon, France, 28–30 November 1990 World Health Organization Available at: https://iris.who.int/handle/10665/60870.

[ref79] Wu L. Dong Y. Zhu C. Chen Y. (2023). Effect and mechanism of acupuncture on Alzheimer’s disease: a review. Front. Aging Neurosci. 15:1035376. doi: 10.3389/fnagi.2023.1035376, PMID: 36936498 PMC10020224

[ref80] Xie D. P. Zhou G. B. Chen R. L. Qin X. L. Du J. D. Zhang Y.. (2020). Effect of Electroacupuncture at Zusanli (ST36) on Sepsis induced by Cecal ligation puncture and its relevance to spleen. Evid. Based Complement. Alternat. Med. 2020, 1–10. doi: 10.1155/2020/1914031, PMID: 33082818 PMC7563055

[ref82] Xu J. Zheng X. Cheng K.-K. Chang X. Shen G. Liu M.. (2017). NMR-based metabolomics reveals alterations of electro-acupuncture stimulations on chronic atrophic gastritis rats. Sci. Rep. 7:45580. doi: 10.1038/srep45580, PMID: 28358020 PMC5372362

[ref81] Xu Z. Li R. Zhu C. Li M. (2013). Effect of acupuncture treatment for weight loss on gut Flora in patients with simple obesity. Acupunct. Med. 31, 116–117. doi: 10.1136/acupmed-2012-010209, PMID: 22961606

[ref83] Yamamoto H. Kawada T. Kamiya A. Miyazaki S. Sugimachi M. (2011). Involvement of the mechanoreceptors in the sensory mechanisms of manual and electrical acupuncture. Auton. Neurosci. 160, 27–31. doi: 10.1016/j.autneu.2010.11.004, PMID: 21167796

[ref84] Yang N.-N. Ye Y. Tian Z.-X. Ma S.-M. Zheng Y. Huang J.. (2020). Effects of Electroacupuncture on the intestinal motility and local inflammation are modulated by Acupoint selection and stimulation frequency in postoperative ileus mice. Neurogastroenterol. Motil. 32:e13808. doi: 10.1111/nmo.13808, PMID: 32114712

[ref85] Yin Z. Li Y. Zhang X. Xia M. Chen Z. Zhao L.. (2022). Moxibustion ameliorates cognitive function in older adults with mild cognitive impairment: a systematic review and Meta-analysis of randomized controlled clinical trials. Eur. J. Integr. Med. 53:102133. doi: 10.1016/j.eujim.2022.102133

[ref88] Yu J. Min D. Bai Y. Long Q. Zou T. Wang S. (2020). Electroacupuncture alleviates Parkinson disease and regulates the expression of brain-gut peptides. Exp. Anim. 69, 448–460. doi: 10.1538/expanimgerm.19-0153, PMID: 32669479 PMC7677085

[ref86] Yu Z. (2020). Neuromechanism of acupuncture regulating gastrointestinal motility. World J. Gastroenterol. 26, 3182–3200. doi: 10.3748/wjg.v26.i23.3182, PMID: 32684734 PMC7336328

[ref87] Yu Z. Lu Luo Y. Li Q. W. Deng S. Lian S. Liang F. (2014). Different manual manipulations and electrical parameters exert different therapeutic effects of acupuncture. J. Tradit. Chin. Med. 34, 754–758. doi: 10.1016/S0254-6272(15)30092-3, PMID: 25618982

[ref91] Zhang B. Han Y. Huang X. Liu Z. Li S. Chang J.. (2019). Acupuncture is effective in improving functional communication in post-stroke aphasia: a systematic review and Meta-analysis of randomized controlled trials. Wien. Klin. Wochenschr. 131, 221–232. doi: 10.1007/s00508-019-1478-5, PMID: 31001680

[ref90] Zhang J. Chunjian L. Xiaoxiong W. Nie D. Haibo Y. (2021). Neuroplasticity of acupuncture for stroke: An evidence-based review of MRI. Neural Plast. 2021, 1–14. doi: 10.1155/2021/2662585, PMID: 34456996 PMC8397547

[ref89] Zhang Z. Chen L. Guo Y. Li D. Zhang J. Liu L.. (2023). The neuroprotective and neural circuit mechanisms of Acupoint stimulation for cognitive impairment. Chin. Med. 18:8. doi: 10.1186/s13020-023-00707-x, PMID: 36670425 PMC9863122

[ref92] Zhang Z. Sui R. Ge L. Xia D. (2022). Moxibustion exhibits therapeutic effects on spinal cord injury via modulating microbiota Dysbiosis and macrophage polarization. Aging 14, 5800–5811. doi: 10.18632/aging.204184, PMID: 35876627 PMC9365548

[ref93] Zhao Z.-Q. (2008). Neural mechanism underlying acupuncture analgesia. Progr. Neurobiol. 85, 355–375. doi: 10.1016/j.pneurobio.2008.05.004 18582529

