# Supplementary material for: Acupuncture and moxibustion therapy for cognitive impairment: the microbiome–gut–brain axis and its role
Source: Front Neurosci. 2024 Jan 11;17:1275860. doi: 10.3389/fnins.2023.1275860 (PMC10808604; doi:10.3389/fnins.2023.1275860)
Supplement: Supplementary file 1 [file Data_Sheet_1.docx]

Supplementary Material

# Supplementary Figures and Tables

## Supplementary Figures

##
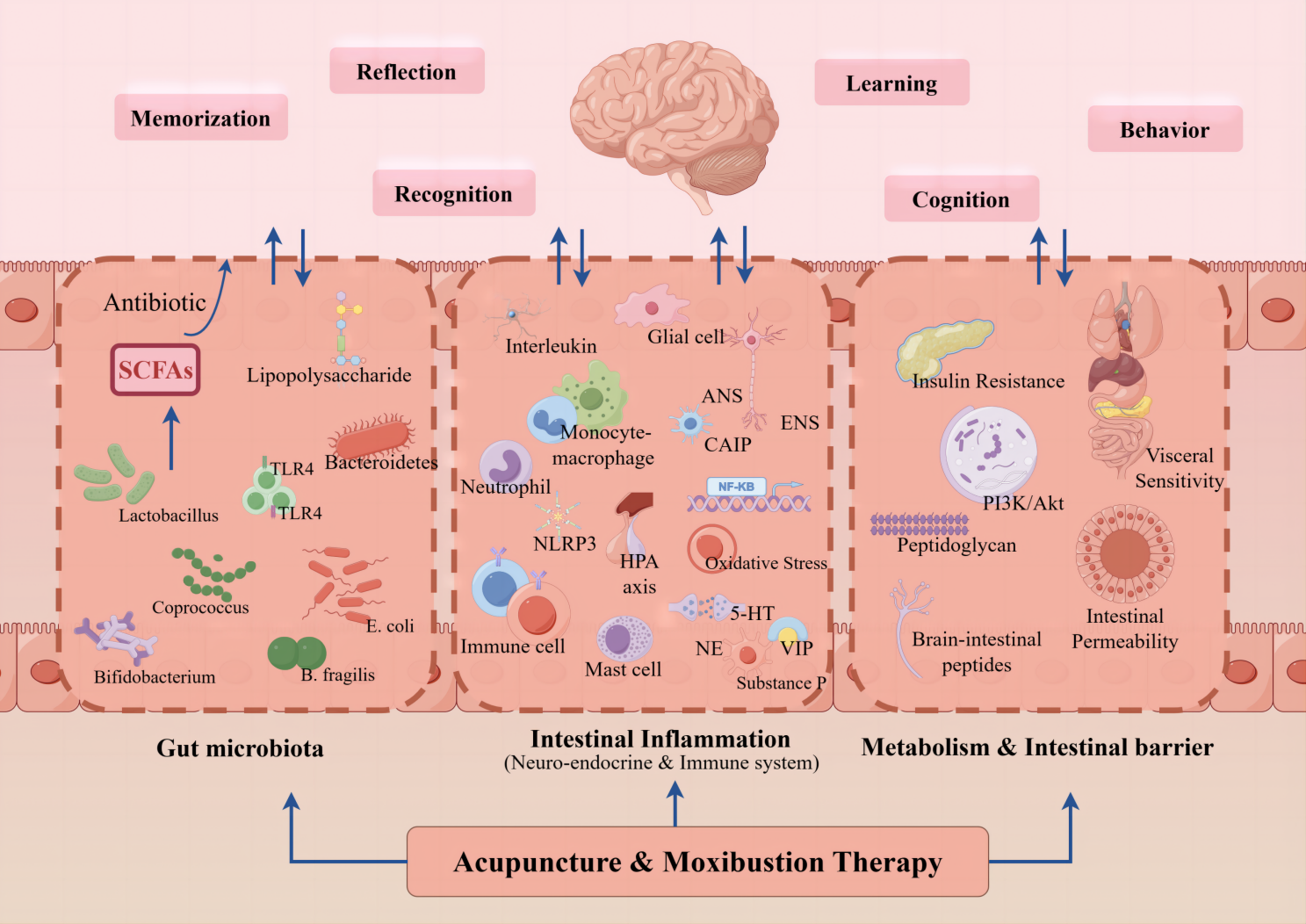


## **Supplementary Figure 1.** The role of acupuncture and moxibustion therapy on cognition through the MGB axis. The therapy could improve cognition in three major ways. Firstly, it restores the diversity and compositions of gut microbiota to improve cognition via promoting anti-inflammatory probiotics and inhibiting pro-inflammatory pathogenic bacteria. Secondly, it reduces intestinal inflammation mediated by Neuro-endocrine and Immune system, involving immune cells, monocyte-macrophage system, neuropeptides and neurotransmitters, oxidative stress, HPA axis and multiple neural pathways so as to ameliorate central inflammation and neuronal death. Thirdly, it regulates metabolism and intestinal barrier to reduce visceral sensitivity and intestinal permeability to prevent the absorption of inflammatory substances. Figure created using Figdraw (www.figdraw.com). TLR4, Toll-Like Receptor 4; E. coli, Escherichia coli; CNS, central nervous system; ANS, autonomic nervous system; SCFAs, short-chain fatty acids; HPA axis, Hypothalamic-Pituitary-Adrenal Axis; NE, norepinephrine; VIP, vasoactive intestinal peptide; 5-HT, 5-hydroxytryptamine; CAIP, Cholinergic anti-inflammatory pathway.

## Supplementary Tables

| Table 1 Experimental and clinical data revealing mechanisms of acupuncture points to regulate Microbiome-gut-brain axis | | | | | |
| --- | --- | --- | --- | --- | --- |
| Intervention | Acupoint | Parameters | Model/participants | Mechanisms/Improvement index | Ref. |
| EA & Moxibustion | Bilateral ST36 and CV4 | - | DSS-induced UC mice | Balance the major communities (inhibiting pathogens and promoting probiotics) through improving the alpha diversity indices and beta diversity distributions of intestinal flora | [79] |
| Moxibustion | Bilateral ST25 and RN6 | - | Rat model of UC | Down-regulate the expression of TGF-β1 and IGF-1 to rectify abnormal immune responses Promote gut flora back towards normal by decreasing E. coli and B. fragilis improving the colonic mucosa | [76] |
| EA & Moxibustion | Bilateral ST36 and RN4 | 0.1mA, 2-100Hz, 2-4V | DSS-induced UC mice | Restore the normal ratio of Treg and Th17 cells in spleen lymphocytes of UC mice, improving intestinal inflammatory response and rebuilding intestinal immune balance | [72] |
| Moxibustion | Bilateral ST25 | - | Mice treated with a surgical procedure to induce SCI | Mitigate decrease of gut bacterial, improve locomotor recovery Down-regulate expression of IL-1β, IL-17, IFN-γ Up-regulate tight junction protein to reduce the apoptosis of epithelial cells | [92] |
| EA | Bilateral BL60 | 2 Hz, 2 mA | Rat model of adjuvant arthritis | Down-regulate the expression of TLR4, MyD88, and NF-κB | [15] |
| EA | Bilateral ST36 and ST 37 | 2 Hz, 1 mA | TNBS-induced colitis rats | Relieve visceral hypersensitivity by inhibiting TH expression in the sixth lumbar dorsal root ganglia, restoring local inflammatory damage | [77] |
| (continued on next page) | | | | | |
| Table 1 (continued) | | | | | |
| Intervention | Acupoint | Parameters | Model/participants | Mechanisms/Improvement index | Ref. |
| EA | Bilateral ST36, LI11 and ST25 | 2 V, 3 Hz | A sepsis rat model induced by CLP | Inhibit intestinal permeability, alleviate inflammation reaction, exerting protective effects on intestinal barrier in septic rats Decrease the levels of TNF-α, IL-10 and D-LA in serum and normalize intestinal T-cell immunity | [84] |
| EA | Bilateral ST36 | 1.0 mA and 2 Hz | DSS-induced colitis mice | Up-regulate cadherin, claudin-1 and zonula occludens-1 levels Increase adiponectin, maintain mucosal tight junctions, modulate gut microbiota by TLR4 signaling via MyD88-dependent pathway | [41] |
| EA | Bilateral ST25, ST36 | 1 mA,2, 10, 30, and 100 Hz | Intestinal manipulation procedure-induced POI mice | Inhibite activity of MPO produced by granulocytes and immune cells infiltration  Increase the gastrointestinal motility and attenuate peripheral inflammation | [88] |
| MA | Bilateral ST36 | - | CCH rats | Activate the JAK2/STAT3 pathway(α7nAChR downstream pathways)  Reduce expression of TNF-α and IL-6 in the hippocampus to protect neurons | [8] |
| EA | Bilateral ST25, ST36 | 2/15 Hz, 0.8 ~ 1.3 mA / | Rat model of D-IBS | Attenuate changes of 5-HT, CGRP and NPY in GBA in the rat model of D-IBS to a normal state, attenuating visceral sensitivity | [53] |
| EA | Bilateral ST25, SP15 | 15 Hz/10 mA | Patients diagnosed with T2DM | Improve insulin sensitivity and T2DM through various mechanisms, such as anti-inflammatory, and the improvement of lipid metabolism and adipokines | [20] |
| (continued on next page) | | | | | |
| Table 1 (continued) | | | | | |
| Intervention | Acupoint | Parameters | Model/participants | Mechanisms/Improvement index | Ref. |
| EA | Bilateral ST36 | 1 V, 1 Hz, | Endotoxemia in mice induced by LPS from Escherichia coli | Decrease TNF-α production in the spleen via activation of the vagus nerve and splenic nerve activity originating from the DVC | [42] |
| Moxibustion | Bilateral ST25 | - | TNBS-induced colitis rat | Increase the degranulation of Mast Cell, release bioactive mediators (e.g. Histamine, SP, 5-HT) to exert healing effect on colonic mucosal damage | [90] |
| EA | Bilateral ST2, ST21, ST36 | 4 Hz / 50 Hz, 2 to 4 V | Rat model of CAG | Restore perturbation in gut microbiota metabolism like serum β-glucose and inositol in CAG rats. Relieve gastric mucosal damage and enhance the regularity of gastric myoelectrical activities via regulating the content of gastrin, SP and EGF | [86] |
| EA | Bilateral PC6, ST36, and SP6 | sparse wave 2 Hz, dense wave 50 Hz | OLETF rats | Reduce the insulin resistance of rats by restoring the level of insulin signaling related molecules like IRS-1, IRS-2 and Akt2 to normal and activating the signal transduction pathway of the phosphatidylinositol 3-kinase (PI3K)/Akt | [28] |
| EA, electroacupuncture; DSS-induced, dextran sulphate sodium-induced; UC, ulcerative colitis; D-IBS, diarrheal irritable bowel syndrome; CCH, Chronic cerebral hypoperfusion; CLP, cecal ligation and puncture; OLETF, Otsuka Long-Evans Tokushima Fatty; TH, tyrosine hydroxylase; DVC, dorsal vagal complex; EGF, epidermal growth factor; MPO, myeloperoxidase; MA, manual acupuncture; 5-HT, 5-hydroxytryptamine; SP, substance P; VIP, vasoactive intestinal peptide; p-ERK1/2, extracellular signal-regulated kinase1/2 protein. | | | | | |

| Table 2 The effects of acupuncture-moxibustion treatment specific microbiota species | | | | |
| --- | --- | --- | --- | --- |
| Model | | Intervention | Microbiota species | Study |
| Patients with Knee Osteoarthritis | | EA | Increase Bacteroide and Agathobacter Reduce Streptococcus | [72] |
| UC model rats | | EA | Increase Lactobacillus and Spirillum Reduce Clostridium bicarbonate | [43] |
| DSS-induced chronic colitis rats | | EA | Up-regulate Bacteroidetes, Muribaculaceae, Faecalibacterium, Roseburia, and Bifidobacterium Down-regulate Firmicutes, Proteobacteria, Escherichia-Shigella, and Erysipelatoclostridium | [69] |
| DSS-induced colitis mice | | EA | Up-regulate Lactobacillaceae Down-regulate Turicibacteraceae, Clostridiaceae, and Erysipelotrichaceae | [39] |
| Patients with mild to moderate active CD and poor response to drugs | | MA | Up-regulate SCFAs producing bacteria (Lachnospira, Coprococcus, Roseburia and Roseburia faecis) and anti-inflammatory bacteria (Faecalibacterium and F. prausnitzii) | [4] |
| Ulcerative colitis rats | | EA | Increase Lachnospiraceae bacterium and Lactobacillus species Decrease Clostridium bifermentans | [63] |
| Patients with simple obesity | | MA | Increase Lactobacillus and Bifidobacterium Decrease Bacteroides and Clostridium perfringens | [80] |
| Mouse Model of PD | | MA | Increase Butyricimonas Reduce genus Bacteroides (Bacteroides fragilis) | [27] |
| (continued on next page) | | | | |
| Table 2 (continued) | | | | |
| Model | | Intervention | Microbiota species | Study |
| DSS-induced colitis mice | | EA | Restore Firmicutes (Lachnospiraceae, Proteobacteria and Enterobacteriaceae) | [41] |
| Elder rats | | Moxibustion | Increase intestinal probiotics (mainly Lactobacillus) and Firmicute Decrease Bacteroidetes | [49] |
| DSS-induced colitis rats | | Moxibustion | Lower Proteobacteria and the genera Saccharibacteria, Sphingomonas and Barnesiella | [52] |

EA, electroacupuncture; DSS-induced, dextran sulphate sodium-induced; UC, ulcerative colitis; MA, manual acupuncture; PD, Parkinson's disease.
